# Providing dignity therapy to patients with advanced cancer: a feasibility study within the setting of a hospital palliative care unit

**DOI:** 10.1186/s12904-021-00821-3

**Published:** 2021-08-16

**Authors:** Francesca Nunziante, Silvia Tanzi, Sara Alquati, Cristina Autelitano, Enrica Bedeschi, Elisabetta Bertocchi, Matilde Dragani, Davide Simonazzi, Elena Turola, Luca Braglia, Luciano Masini, Silvia Di Leo

**Affiliations:** 1 Medical Oncology Department, Azienda USL-IRCCS di Reggio Emilia, Reggio Emilia, Italy; 2 Palliative Care Unit, Azienda USL-IRCCS di Reggio Emilia, Reggio Emilia, Italy; 3 Clinical and Experimental Medicine PhD Program, University of Modena and Reggio Emilia, Modena, Italy; 4 Rheumatology-Diabetology Week Hospital, Azienda USL-IRCCS di Reggio Emilia, Reggio Emilia, Italy; 5 Primary Care, Azienda USL–IRCCS di Reggio Emilia, Reggio Emilia, Italy; 6 Scientific Directorate, Azienda USL-IRCCS di Reggio Emilia, Reggio Emilia, Italy; 7 Research and Statistics Infrastructure, Azienda USL-IRCCS di Reggio Emilia, Reggio Emilia, Italy; 8 Department of Medicine and Long-Term Care, Casa di cura Villa Verde, Reggio Emilia, Italy; 9 Psycho-Oncology Unit, Azienda USL-IRCCS di Reggio Emilia, Reggio Emilia, Italy

**Keywords:** Dignity Therapy, Palliative care, Cancer, Nurses, Feasibility studies, Mixed-method study, Dignity-related distress

## Abstract

**Background:**

Dignity is a basic principle of palliative care and is intrinsic in the daily practice of professionals assisting individuals with incurable diseases. Dignity Therapy (DT) is a short-term intervention aimed at improving the sense of purpose, meaning and self-worth and at reducing the existential distress of patients facing advanced illness. Few studies have examined how DT works in countries of non-Anglo Saxon culture and in different real-life settings. Moreover, most studies do not provide detailed information on how DT is conducted, limiting a reliable assessment of DT protocol application and of its evaluation procedure.

The aim of this study was to assess the feasibility and acceptability of a nurse-led DT intervention in advanced cancer patients receiving palliative care.

**Method:**

This is a mixed-method study using before and after evaluation and semistructured interviews. Cancer patients referred to a hospital palliative care unit were recruited and provided with DT. The duration of sessions, and timeframes concerning each step of the study, were recorded, and descriptive statistical analyses were performed.

The patients' dignity-related distress and feedback toward the intervention were assessed through the Patient Dignity Inventory and the Dignity Therapy Patient Feedback Questionnaire, respectively. Three nurses were interviewed on their experience in delivering the intervention, and the data were analyzed qualitatively.

**Results:**

A total of 37/50 patients were enrolled (74.0%), of whom 28 (75.7%) completed the assessment. In 76.7% of cases, patients completed the intervention in the time limit scheduled in the study. No statistically significant reduction in the Patient Dignity Inventory scores was observed at the end of the intervention; most patients found DT to be helpful and satisfactory. Building opportunities for personal growth and providing holistic care emerged among the facilitators to DT implementation. Nurses also highlighted too great of a time commitment and a difficult collaboration with ward colleagues among the barriers.

**Conclusions:**

Our findings strongly support the acceptability, but only partially support the feasibility, of nurse-led DT in advanced cancer patients in a hospital setting. Further research is needed on how to transfer the potential benefits of DT into clinical practice.

**Trial registration:**

Retrospectively registered on ClinicalTrial.gov NCT04738305.

## Background

First described by Chochinov in 2002 [1] as a short-term intervention aimed at reducing psycho-emotional and existential distress and improving the sense of personhood, purpose, meaning and self-worth of patients facing advanced illness, Dignity Therapy (DT) engages individuals in a contemplation of their life experience and the aspects that they consider most important and that they wish to share. Dignity is a central tenet to palliative care and is intrinsic to its daily practice, from dealing with symptom management and psychosocial and spiritual well-being, to caring for patients and their family as a whole; it is also a constant driver of healthcare professionals assisting individuals with an incurable illness, particularly of nurses who spend most time with them. Nurses’ role in preserving this domain, potentially enriching or damaging a patient’s sense of dignity, is highlighted in several documents [2–6] governing the profession worldwide. In literature, the multifaceted aspects of dignity are broadly classified into three categories, namely, intrinsic dignity, subjective dignity, and relational dignity [7]. Each of these definitions has its own limits, and none alone can be an exhaustive premise for nurturing both the bioethical debate and clinical practice, especially with reference to palliative care. In recent years, many groups have focused on ways to assure that dignity preservation become part of routine care by implementing DT interventions, but there is still little evidence about the best way to conduct this and measure its impact. As concluded in the systematic review by Martinez et al. [8] on DT outcomes in patients with advanced life-threatening diseases, although findings on its efficacy are inconsistent, patients and relatives report helpfulness and meaningfulness. Similar findings have been reported by Xiao et al. [9] in their review on cancer patients in palliative care. The lack of evidence of efficacy could be due not only to the distance between DT effects and the outcome measures, but also to differences in the settings and cultural environment where it has been implemented. Most studies do not provide detailed information on how DT was conducted, limiting a complete and reliable assessment of DT protocol applications and its evaluation procedures [8, 9]. Moreover, DT has been largely studied in Anglo-Saxon cultures; therefore, it is likely that its application in sociocultural contexts with different values requires some adjustments [10–16]. Studies on DT in Italy are poor and focused on its application by trained psychotherapists [17, 18]. Current evidence suggests the need to investigate how DT can comprehensively work in different cultural contexts and real-life settings, to discover the time and resource commitments required to deliver DT and to explore the experience of professionals delivering it [9, 19–24].

The present work aims to assess the feasibility and acceptability of nurse-led DT intervention in advanced cancer patients receiving palliative care in a hospital setting in Italy.

The specific aims were as follows:

- assessing the patients' sense of dignity before and after the intervention
- assessing the patients’ feedback toward the intervention
- exploring the experiences of nurses involved in the DT implementation process.

## Methods

### Study design

This is a mixed method study using a before and after evaluation and semistructured interviews [25–27]. It consists of quantitative and qualitative data collection and analysis and a final triangulation of the results.

### Setting

The study was implemented within the Palliative Care Unit (PCU) and the Medical Oncology Department of Santa Maria Nuova Hospital. This is a public research hospital with 900 beds, accredited as a Clinical Cancer Centre by the Organization of European Cancer Institutes (OECI). The Palliative Care Unit (PCU) is a specialized hospital-based unit with no beds that was established in April 2013 with the mission of delivering clinical, research and training activity in this field. PCU members address clinical consultations both to inpatients with advanced illness from all hospital wards and to outpatients in charge of the hospital. The Medical Oncology Department is a 20-bed unit for patients with cancer or hematological malignancies suffering from complications related to antineoplastic treatment or disease progression. Medical Oncology and PCU work closely together on a daily basis in a collaborative approach.

### Study population

Fifty advanced cancer patients with over 3 months of life expectancy, according to physicians' judgment, that were referred to the PCU were screened for eligibility during a palliative care consultation. Eligibility criteria included: age 18 years or more, a performance status (measured with the Eastern Cooperative Oncology Group-ECOG) between 0 and 2, awareness of being affected by an incurable cancer and the cognitive ability (according to physicians' judgment) to read, understand, and fill in a questionnaire.

### Assessment and intervention procedures

A PCU physician identified eligible patients over a six-month period. DT intervention was conducted by five nurses of the Medical Oncology Department, who had basic knowledge of palliative care and were routinely involved in hospitalized cancer patient care.. The choice of having nurses deliver DT was based on the literature [28, 29] and in-depth theoretical reflections conducted by the principal investigator of this study (a nurse previously trained on DT). Nurses had been preliminarily trained on DT intervention by a PCU physician and a psychologist expert in palliative care. We developed an intervention guide for DT implementation, including instructions for introducing the DT interview and providing information throughout the entire process.

The Italian version of the DT Question Protocol was employed [30] (Table 1, page 28). Nurses conducted the interview following a core collection of questions not previously delivered to patients. The interviews were audio-recorded and transcribed, and the transcripts were revised to improve the syntax (spoken to written language). The patients read the revised transcript, and their feedback was considered to develop the final “Generativity Document”, i.e., the written legacy focusing on what the meaning of life is for that patient, the most important messages he/she wants to leave to his/her loved ones and the way he/she would like to be remembered by them[1]. The Generativity Document was delivered to the patient, who could then share it with his/her family or friends. Throughout the study, DT nurses participated in weekly sessions held by PCU members and by the psychologist concerning emotional and communicative issues management.

**Table 1:** English and Italian versions of the Dignity Therapy Question Protocol

dignity therapy question protocol [31]	domande del protocollo della terapia della dignità [30]
1. Tell me a little about your life history; particularly the parts that you either remember most or think are the most important? When did you feel most alive?	1. Mi racconti qualcosa della sua vita: quali sono le parti che ricorda di più o che pensa siano per Lei più importanti? Quando si è sentito/a più vivo/a?
2. Are there specific things that you would want your family to know about you, and are there particular things you would want them to remember?	2. Ci sono delle cose della sua vita che Lei vorrebbe che la sua famiglia o persone per Lei significative sapessero di Lei o cose particolari che vorrebbe ricordassero?
3. What are the most important roles you have played in life (family roles, vocational roles, community-service roles, etc.)? Why were they so important to you, and what do you think you accomplished in those roles?	3. Quali sono stati i ruoli più importanti che Lei ha avuto nella sua vita (in famiglia/nel lavoro/in società) e che cosa pensa di aver realizzato in quei ruoli?
4. What are your most important accomplishments, and what do you feel most proud of?	4. Quali sono le cose più importanti che ha realizzato nella sua vita e di che cosa si sente più orgoglioso?
5. Are there particular things that you feel still need to be said to your loved ones or things that you would want to take the time to say once again?	5. Ci sono cose che Lei non ha mai detto e sente di voler dire ai suoi cari o cose che Lei vorrebbe avere il tempo di dire ancora una volta?
6. What are your hopes and dreams for your loved ones?	6. Che cosa spera e che cosa desidera per i suoi cari?
7. What have you learned about life that you would want to pass along to others? What advice or words of guidance would you wish to pass along to your (son, daughter, husband, wife, parents, other[s])?	7. Quali sono le cose che ha imparato sulla vita che vorrebbe trasmettere agli altri? Quali consigli o parole che li orientino vorrebbe trasmettere ai suoi figli/marito/moglie/genitori o altre persone per Lei significative?
8. Are there words or perhaps even instructions that you would like to offer your family to help prepare them for the future?	8. Ci sono parole o consigli che Le piacerebbe offrire alla sua famiglia per aiutarla a prepararsi per il futuro?
9. In creating this permanent record, are there other things that you would like included?	9. Nel creare questo documento permanente, ci sono altre cose che Le piacerebbe includere?

**Fig. 1:**
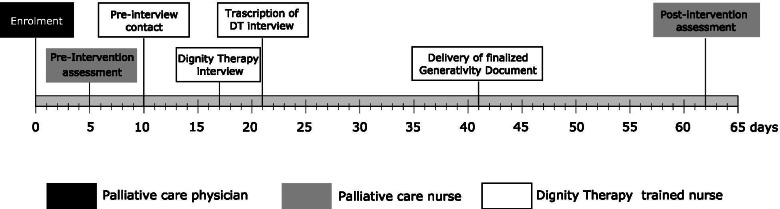
Dignity Therapy intervention procedures. Patients who gave their written informed consent to participate went through the pre-intervention assessment by means of a self-administered questionnaire. Within the following 5 days, they were phoned by a DT-trained nurse to schedule a vis-a-vis encounter, which took place 3 to 7 days later. During this encounter, they were provided with further information about the intervention, and the DT interview was administered. In contrast to the original protocol, DT questions were not delivered in advanced to subjects. DT interviews were audio-recorded and transcribed verbatim within 4 days. In the next 3 weeks, the transcription was read and discussed together with the subject, who could suggest any modification or integration. The implementation process, which could include subsequent encounters, ended when the final version of the Generativity Document was finalized and delivered to the patient. Finally, 2 or 3 weeks later, subjects were phoned by another nurse to perform the post-intervention evaluation by means of two self-administered questionnaires

Each step of the planned study process and any deviation from the established standards were documented in detail. The study procedures and timeframes scheduled for each step are reported on in Fig. 1.

### Outcome measurements

DT feasibility and acceptability were assessed by measuring the enrollment and retention rates, the duration of each DT session and the timeframes concerning each study step. Patient dignity-related distress was assessed before and after the DT intervention through the Italian version of the Patient Dignity Inventory (PDI) [32]. This is a valid and reliable 25-item, self-administered questionnaire aimed at investigating dignity-related issues known to affect the sense of dignity of advanced cancer patients. It measures on a 5-point Likert scale the perceived distress in the major dignity categories: illness-related concerns, dignity-conserving repertoire and social dignity inventory [33]. PDI has been translated and validated in Italian [32]The one-dimensionality of the scale has been demonstrated for the Italian version of the construct.

The patient’s opinion on the DT intervention was assessed at the end of the study through the Dignity Therapy Patient Feedback Questionnaire [34]. It is a 5-point Likert scale, 23-item self-administered questionnaire investigating patients’ views on DT intervention and how this has influenced their lives. The Dignity Therapy Patient Feedback Questionnaire was translated into Italian for our study purpose.

Professionals’ experience in implementing DT was explored through semistructured interviews administered to the three nurses who had performed the highest number of DT interventions. The interview topic guide was developed by a member of the research team with expertise in qualitative evaluation. It concerned motivations and expectations toward the DT intervention, relationships engaged with patients and other professionals within the study, and opinions on DT implementation in clinical practice.

Anonymity and non-traceability criteria were presented to all interviewees. Explicit permission was requested to audio-record the interviews. Two psychologists with a basic knowledge of DT but who were not involved in the study performed the interviews. A nursing student and a trainee psychologist observed and took field notes on nonverbal communication.

### Data analysis

The statistics performed included time spent (mean and confidence interval) performing DT interviews and developing the Generativity Document and pre-post PDI changes (variation analyzed with a paired t-test). The data are expressed in terms of frequency and percentage for categorical variables, mean standard deviation for symmetric quantitative variables, and median + IQR for skewed variables. 95% confidence intervals were calculated by the Clopper-Pearson method for proportions. The tests were two-sided, and p-values < 0.05 were considered statistically significant. The statistical analysis was performed using R 3.3.3 [35].

The qualitative assessment was performed by employing the Framework Analysis [26, 27]. First, two researchers developed coding schemes covering themes in accordance with the assessment focus. They then independently analyzed the transcripts and categorized all the potentially relevant text segments. Third, the two researchers compared categorizations and reconsidered and discussed any differences in interpretation to reach an agreement. The final categorization was discussed and revised with the other members of the research team.

The qualitative evaluation was performed and reported in accordance with the consolidated criteria for reporting qualitative research (COREQ) guidelines [36﻿].

## Results

The following paragraphs report findings from both the quantitative and qualitative analyses performed in this study.

### Quantitative assessment

**Fig. 2:**
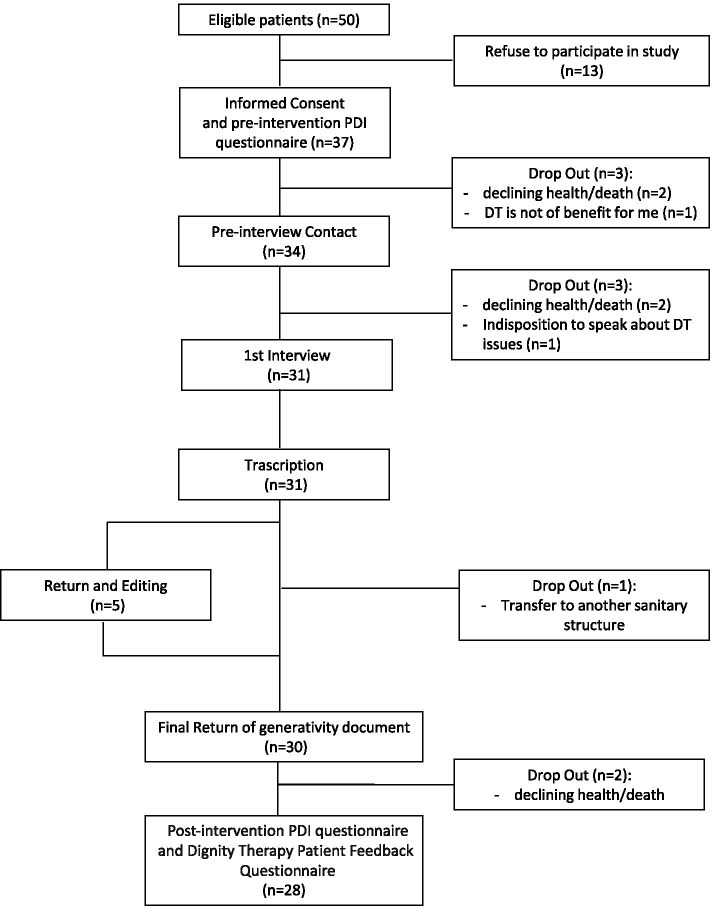
Patient’s flow in the study

Fifty patients referred to the hospital PCU between June 2016 and April 2017 were recruited. Thirteen patients (26.0%, CI 95%: 14.6–40.3) declined participation, with most unwilling to speak about DT issues. Thirty-seven patients participated in the study, with an enrollment rate of 74.0% (CI 95% 59.7–85.4). Twenty-eight of them (75.7%; CI 95%: 58.8– 88.2) completed the DT intervention until the final assessment (Fig. 2). Withdrawal reasons concerned clinical worsening or patient death, as well as unwillingness to speak about DT issues. The participants’ mean age was 65 years (41 to 89). The sample was homogeneous by gender. Most subjects were married and Catholic; half of the sample had a secondary school diploma or a degree. Tumors from the gastrointestinal system, lung or breast accounted for 81.1% of the cancer diagnoses. A total of 70.2% of the cancer diagnoses had been received in the past two years (median 1.17; IRQ 0.04–2.27). Most patients (95.59%) scored between 0 and 1 at the ECOG performance status (Table 2, page 29).

**Table 2:** Sociodemographic and clinical characteristics of enrolled patients

	**Number (n = 37)**
**Sex, M/F**	18/19
**Mean age at diagnosis (± SD)**	62.7 ± 17.8
**Educational Level**	
Primary	3
Lower secondary education	5
Upper secondary education	18
Higher education	9
Not available	2
**Marital Status**	
Unmarried	3
Married	28
Separate/Divorced	4
Widow/er	2
**Religion**	
Catholic	28
Other	8
Not Available	1
**Malignancy**	
Gastrointestinal	13
Lung	12
Breast	5
Bone and soft tissue	2
Hematopoietic and lymphoid tissues	2
Urogenital	2
Neuroendocrine	1
**Performance Status (ECOG)**	
0	25
1	10
2	2

The DT interviews had a mean duration of 85.83 ± 40.09 min. Moreover, nurses spent between 80 and 332 (mean: 152.07 ± 57.19) minutes in each Generativity Document.

In 76.7% of cases (95% CI: 57.7–90.1), the Generative Document was delivered within the time limit scheduled in the study protocol (median: 12 days, range: 7–24).

No statistically significant modification in the overall score or single items of the PDI were observed in the before and after analysis.

The Dignity Therapy Patient Feedback Questionnaire revealed that most patients found DT helpful (82.2%) and satisfactory (92.9%). The majority (82.9%) reported that DT helped them accept the state of things, heightened their sense of dignity (78.6%) and made them feel that their life had more meaning (78.6%). 75.0% stated that DT increased their sense of self-continuity and gave them a sense of looking after unfinished business. A total of 75.0% of participants felt that DT helped their families. Nearly half of the participants reported that DT improved their quality of life and spiritual well-being (57.2%) and lessened suffering (53.6%), sadness or depression (50.0%) and the feeling of being a burden to others (46.4%).

### Qualitative assessment

All three nurses accepted to be interviewed. Interviews took place within the hospital and lasted between 30 and 50 min.

The interviews focused on two main themes, i.e., facilitators and barriers to DT implementation in the hospital setting. Several subthemes were also identified. Moreover, some suggestions emerged to ease the introduction of DT into clinical practice.

Each subtheme was further classified, as related to the nurse personal characteristics (subjective domain), the nurse-patient relationship (relational domain) or the hospital environment (organizational domain).

The following paragraphs describe the subthemes in each domain. Table 3 (page 30) lists the subthemes, together with representative quotations from the participants’ interviews.

**Table 3:** Facilitators and barriers to DT implementation according to the nurses’ experience, and representative quotations from qualitative analysis

	**Facilitators**	**Barriers**
**Subjective domain**	***Sensibility*** *N1: “I discovered in her [the patient] a great deal of humanity that usually did not show, did not transpire and an openness… towards this endless love for people, towards mankind and to the ability…and that I fully share…”*	***Sensibility*** *N3:* “A*t the beginning it was quite tough, especially from a psychological point of view [….] From an organization point of view, it just requires getting things together, but it was challenging from a psychological point of view…”*
	***Motivation*** *N2: “I think someone needs to do it. So, it’s better if we do it, since we’re a bit more incline or at least we’ve got a bit more…drive in doing it, rather than someone else who does so unwillingly…without having chosen it”*	***Feeling unprepared*** *N2:* “*…at the beginning I was a bit scared, I mean I felt a bit… I don’t know how to say this….tiny compared to this huge project, since I was a bit ripe also in terms of experience…”* *N1: “…at the same time I had some things happening in my private life that surely took up additional time…and in terms of emotional energy… So, my main concern was staying on balance on what was happening to me…”*
**Relational domain**	***Promoting awareness of the meaning of one’s life*** *N1: “Though there is one thing I’ve always appreciated about humankind, and that’s that each of us has…their own joys…that are uniquely personal”*	***Engaging too close of a relationship with the patient*** *N1: “In any case there is this moment of intimacy that comes out so strongly … So […] I recall that at times it would be tough…just because of this…”* *N3: “I set in parallel my emotions of my short life up to now, with his [the patient] and I tried to draw a comparison… I don’t know, I put myself in his shoes… a bit difficult to bare…”*
	***Knowing the patient beyond his/her illness*** *N3: “From my point of view it was enriching. Because we professionals give a lot for granted, we only see the patients in their pajamas and we overlook the 60–70 years gone by before they entered the hospital…practically their entire life!”*	
	***Building opportunities for personal growth*** *N2: “I believe it was very useful for the patients, because they had the chance to think about questions we often are unable ask ourselves in life, and that even we healthy people should be asking ourselves …”* *N1: “…I admit he [the patient] moved me, in the way that he made me live over what he had lived […] and reflect on my own experience…”*	
	***Bringing to light existential and private issues*** *N3: ****“****Perhaps some patients were in distress over simple things that could have been easily solved, things that had not been said or other things like that. So, it [DT] helped patients to uncover a lot of things that could also help in the last month or in the last part of life. Not just those who had a month left, but also those who lived longer […] and that had a need for closeness but did not have the courage to ask for it. “*	***Talking about private issues with an unknown person*** *N2: “Though I felt they were a bit hesitant to open up…and open up with a stranger, especially on topics that are strictly personal…”*
	***Providing holistic care*** *N1: I thought that it [the DT] was a useful tool, both as additional communication tool, compared to the typical communication nurses engage with patients addressing dignity, and as a holistic perspective of taking the patient under one’s care…* “	
	***Perceiving the patient’s feeling of gratitude*** *N2: “I saw there was a lot of appreciation for the work being done, that it was a big or very big deal for them. I saw her [the patient] happy and moved by the words she had confided to me, she thanked me…”*	
**Organizational domain**	***Good collaboration between professionals of the study group*** *N2: “We had meetings to do a recap … with SDL [psychologist], even very useful as we would ask ourselves: in what way do ask this? How do you approach it? Do you keep distant…or do you try to establish a conversation… a friendly one… it gave me the tools to improve my interviews…”* *N3: “It happened that the PCU physician told me: ‘This thing that the patient told you is important because it’s the same thing I’m exploring with him at my consultation …’”*	***Difficult collaboration with colleagues not involved in the study*** *N1:*”… *He [the ward nurse] knew I was inside the room doing the interview. He came into the room, like “I need to change the elastomer” because he had the patient’s therapy scheduled at 11… So fine, he had to have it at 11. …So, a person who must absolutely place an elastomer at 11 o’clock, couldn’t possibly have been able to administer DT…”*
		***Too time-demanding intervention*** *N1:* “Y*eah, I mean it takes up a lot of time, since it’s never just an hour, and then with shifts… I mean, for me it’s practically impossible… But sure, I mean you need time for that too, it’s tiring”* *N2: “I usually carried it [DT] out only after my work shift. When I used to finish my shift, I’d ask patients if they were available for the interview…”*
		***Problems in delivering the Generative Document*** *N2: “Many times I asked them [the patients] to come pick up their Document when they were coming here for their other appointments … Coming to the hospital again, would have been tough …”*

#### Perceived facilitators

##### Subjective domain

The nurses’ sensibility emerged as a paramount characteristic for performing a good DT interview. That is, the attitude to fully listen to the patient’s story while letting themselves be completely involved.

*N1: “I discovered in her [the patient] a great deal of humanity … and an openness… towards this endless love for people … that I fully share…”*

Moreover, the nurses recognized that their motivation and willingness to engage in the study were also driving factors.

*N2: “… We’re a bit more incline or at least we’ve got a bit more…drive in doing it, rather than someone else…”.*

##### Relational domain

The DT protocol questions seemed to be suitable for the purpose of enhancing awareness of the person’s life value for both the interviewee and the interviewer.

DT questions allowed the patients to remember positive experiences of their lives and to explore existential issues. From the nurse’s perspective, this represented a valuable opportunity to experience who the patient was before and beyond his/her illness diagnosis and an opportunity to reflect about their own life and personal growth.

*N3: “… Because we only see the patients in their pajamas and we overlook the 60–70 years gone by before they entered the hospital…”*

Nurses also commented on the extent to which this knowledge positively affected the quality of the care delivered to patients.

Existential issues arising from the DT interviews represented a unique chance for the patient to express desires and preferences that could hardly have emerged in the care relationship, allowing them to be fully supported by professionals.

*N3:* ***“*** *[DT] helped patients to uncover a lot of things that could also help in the last month or in the last part of life. Not just those who had a month left, but also those who lived longer … “*

The nurses highlighted the possible benefits of extending DT intervention to patients in all stages of illness, and to propose it as soon as possible throughout the patient journey, with the objective of supporting the patients' awareness of their inner desires and preferences, and promoting their achievement in time.

DT emerged from nurses’ words as a powerful tool, with the potential of making the patient’s care "holistic" and, therefore, more suitable in fulfilling their diverse needs.

The feeling of gratitude shown by patients at the end of the intervention was perceived as facilitating the DT’s success and increasing the nurses’ self-efficacy.

*N2: “I saw her [the patient] happy and moved by the words she had confided to me, she thanked me…”*

##### Organizational domain

Ongoing training, as well as continuative collaboration and coordination between the PCU and the Medical Oncology Department members, allowed all steps of the DT to be fully implemented.

*N2: “We had meetings … we would ask ourselves: in what way do ask this? How do you approach it? … it gave me the tools to improve my interviews…”*

*N3: “The PCU physician told me: ‘This thing that the patient told you is important because it’s the same thing I’m exploring with him at my consultation …’”*

#### Perceived barriers

##### Subjective domain

If sensitivity allowed the nurses to appreciate the intervention, it was also perceived as a barrier. Indeed, their strong emotional involvement with the patient made them feel overwhelmed and uncomfortable. They perceived themselves as lacking the level of expertise needed to deal with advanced cancer patients and with the broad scope of the DT questions, especially with reference to coping with the patients' emotional reactions.

*N2: “…At the beginning …I felt a bit… tiny compared to this huge project, since I was a bit ripe also in terms of experience…”*

A nurse who was experiencing a stressful life event expressed the fear of not being able to perform the DT intervention with the required commitment and availability.

At the end of the interview, all nurses highlighted the extent to which bringing DT into clinical practice requires specific training, both in terms of DT intervention and its theoretical underpinnings, and remarked on the need for skilled, sensitive, and motivated professionals delivering DT.

##### Relational domain

The fear of lacking the skills to adequately address the patients' emotional reactions is associated with the difficulties of experiencing the very close relationship developed throughout the DT.

*N3: “I set in parallel my emotions of my short life … with his [the patient] … I put myself in his shoes… a bit difficult to bare…”*

On the other hand, patients felt uncomfortable talking about private matters with nurses, being unfamiliar people.

*N2: “…They were a bit hesitant to open up… especially on topics that are strictly personal…”*

A nurse proposed adopting procedures from the original protocol, where questions are provided to patients in advance in order to encourage a preliminary reflection about the existential and meaning issues addressed.

##### Organizational domain

On some occasions, DT interview sessions were interrupted by other nurses who were not aware of the study or who did not recognize DT therapeutic value.

*N1: “… he [the ward nurse] knew I was inside the room doing the interview. He came into the room, … “I need to change the elastomer”…”*

Nurses reported that DT intervention was too time-demanding without a financial reward and viewed this as a sign of a lack of recognition of DT value by the institution.

*N1: “… it takes up a lot of time, since it’s never just an hour, and then with shifts…”*

A nurse proposed relieving professionals implementing the DT of the time-demanding task of transcribing interviews, entrusting this task to others and/or to identify professionals who would perform DT intervention.

In addition, they reported difficulties in delivering the Generativity Documents to patients due to other commitments, such as attending clinical consultations and undergoing therapies. A nurse suggested optimizing patients' time commitment for hospital consultations and treatments in order to deliver the Generativity Document to them.

## Discussion

The present study evaluated the feasibility and acceptability of DT intervention in an Italian setting of cancer patients referred to a hospital PCU, both from the patient’s and healthcare professional’s perspectives.

Our findings strongly support the acceptability of the intervention, but only partially support its feasibility.

On average, more than half of the patients were willing to engage in DT and appreciated the opportunity given to explore the interview’s specific items. One-fourth of the recruited subjects declined, and another fourth did not complete the intervention and/or the after evaluation due to clinical deterioration or death, which is in agreement with most studies on DT [8, 9, 24, 28].

Intervention procedures and assessments were designed considering the evidence and limitations available in the literature, addressing the lack of detailed information about DT conduction and DT therapists’ background and skills.

In general, DT timing has emerged as a relevant aspect. Despite the willingness and efforts that nurses invested in delivering DT, the patients’ precarious health conditions suggest proposing DT at an earlier phase of the disease. Moreover, the time requested for the entire process was considerable, although less than that spent in studies where DT required 3–7 sessions per patient in addition to transcriptions and editing time [23, 34, 37–39].

As reported by the nurses, in most cases the intervention steps were accomplished within the time limits only due to their availability to devote extra time and effort to the study. As a lack of institutional resource is a limit to DT implementation, an abbreviated and less resource-intensive version of DT has been developed and piloted in US [40].

In our study we did not detect any relevant difference in the patients' dignity-related distress between pre to post-intervention, nor with single domains of this construct. Moreover, only half of the subjects who completed the evaluation reported a positive influence of DT on their emotional and spiritual wellbeing, as well as on their quality of life and the perception of not being a burden. On the other hand, DT was highly acceptable to patients, with most of them reporting that it was helpful and satisfactory. These findings mirror those from previous randomized controlled trials [34, 41, 42], quasi-experimental and feasibility studies on DT [11, 14, 16, 19, 20, 23, 41, 43–48] with oncological, respiratory and neurological patients. We agree with previous works’ authors hypothesizing that inconsistent evidence for DT efficacy may partly stem from a lack of specific outcome measures. To overcome this problem, researchers are developing new measurements for the dignity impact that seem to be more consistent with key DT aspects: meaning making, preparation for death and life completion tasks [49].

Implementing DT stimulated considerable proposals on ways to improve it, aimed at reducing the dropout due to clinical deterioration and to optimize the DT's perceived benefits for patients and relatives; among these, the opportunity to also extend its use to patients at the early stages of illness trajectory has been mentioned, an issue that has been explored by studies on cancer patients undergoing chemotherapy [48] and on hematological patients undergoing bone marrow transplantation [50].

The benefits of DT documented in our study concern not only patients receiving the intervention, but also nurses delivering it, who highlighted the value of DT implementation in terms of both personal and professional growth, an issue also reported by others [20, 21].

### Limitations

Our findings need to be interpreted considering some limitations. This is a feasibility study performed on a relatively small sample of subjects. Nevertheless, it allowed us to gather a wealth of information (both quantitative and qualitative) from both intervention users and providers.

Additionally, this was a monocentric study within a specific setting. This allowed the PCU staff to closely manage all phases, from recruitment to evaluation, preparatory training and ongoing DT nurse supervision, and to maintain consistent and complete documentation throughout. However, this also limits the generalizability of our findings to other settings and/or diseases.

## Conclusion

The findings from our study strongly support the acceptability of nurse-led DT in advanced cancer patients in a hospital setting, but only partially support its feasibility.

DT helped patients find a sense of purpose, continuity of self and connection with their family. Its implementation by trained nurses could promote both their personal and professional growth, and potentially improve the quality of care delivered to patients, with reference to the holistic approach underpinning the DT protocol. Nevertheless, feasibility is limited by professionals' time constraints and a lack of recognition of DT value by the institution.

Further research is needed on concealing the time constraints and the lack of resources, with the possibility of transferring potential benefits of DT into clinical practice.

## Data Availability

The study data are securely stored under lock and key at Azienda USL-IRCCS di Reggio Emilia. We do not have permission from the Ethical Committee to release or share the data; thus, we cannot make it available in the public domain. However, the datasets used and analyzed during the current study are available from the corresponding author upon reasonable request.

## Abbreviations

DT: Dignity Therapy
PCU: Palliative Care Unit
PDI: Patient Dignity Inventory
